# Bimagnon dispersion of La_2_CuO_4_ probed by resonant inelastic X-ray scattering

**DOI:** 10.1038/s41598-025-15435-5

**Published:** 2025-10-01

**Authors:** A. Singh, H. Y. Huang, K. Tsutsui, T. Tohyama, S. Komiya, J. Okamoto, C. T. Chen, A. Fujimori, D. J. Huang

**Affiliations:** 1 National Synchrotron Radiation Research Center, Hsinchu, 30076 Taiwan; 2 Department of Physics and Astrophysics, University of Delhi, New Delhi, 110007 India; 3 Synchrotron Radiation Research Center, National Institutes for Quantum Science and Technology, Hyogo, 679-5148 Japan; 4 Department of Applied Physics, Tokyo University of Science, Tokyo, 125-8585 Japan; 5 Central Research Institute of Electric Power Industry, Yokosuka, Kanagawa 240-0196 Japan; 6 Center for Quantum Science and Technology and Department of Physics, National Tsing Hua University, Hsinchu, 30013 Taiwan; 7 Department of Physics, University of Tokyo, Bunkyo-ku, Tokyo, 113-0033 Japan; 8 Department of Physics, National Tsing Hua University, Hsinchu, 30013 Taiwan; 9 Department of Electrophysics, National Yang Ming Chiao Tung University, Hsinchu, 30093 Taiwan

**Keywords:** Superconducting properties and materials, Magnetic properties and materials

## Abstract

We report on the study of the magnetic excitations of Mott insulator $\hbox {La}_{2}\hbox {CuO}_4$ by using resonant inelastic x-ray scattering (RIXS) and cluster calculations within the framework of exact diagonalization. Our results demonstrate experimentally that the bimagnon excitation in Cu $L_3$-edge RIXS is enhanced if the incident x-ray energy is slightly above the absorption edge. Through incident-energy-dependent momentum-resolved RIXS, we investigated the excitation of the bimagnon with predominantly $A_{1g}$ symmetry. The bimagnons of $\hbox {La}_{2}\hbox {CuO}_4$ exhibit a nearly flat dispersion with momentum along the Cu-O bond direction. This observation agrees with the bimagnon dispersion from the calculations on a single-band Hubbard model rather than a Heisenberg model with only the nearest neighbor exchange interaction. This means that the effect of the higher-order spin couplings such as the cyclic or ring exchange interactions caused by the coherent motion of electrons beyond nearest-neighbor sites is important for understanding the bimagnon dynamics of cuprates.

## Introduction

While the mystery of novel cuprate superconductivity is unresolved, spin fluctuations are still considered to play an important role in its pairing mechanism. Spin fluctuations around the antiferromagnetic wave vector could contribute to the pairing interaction^1–6^. The interplay between spin and charge degrees of freedom could be the key to resolving the mystery^6,7^. Understanding the two-dimensional magnetic properties of its mother compound is crucial to describing the metallic behavior of a doped cuprate^8–10^. The Heisenberg model with only the nearest-neighbor exchange interaction *J* is a conventional starting point for the two-dimensional spin-$\frac{1}{2}$ antiferromagnet^11^. This simple model, however, gives reduced spectral weight of magnons along the antiferromagnetic zone boundary^12,13^, and corrections with long-range exchange interactions^12,14^ or higher-order exchange interactions such as ring exchange^12^ are needed. In contrast, the Hubbard model, which comprises the on-site Coulomb energy *U* and the hopping energy *t* between nearest-neighbor Cu sites, is expected to naturally include the effect of such higher-order interactions through the coherent motion of electrons beyond nearest-neighbor sites^15–17^.

There have been extensive studies on the spin fluctuations of undoped cuprates such as $\hbox {La}_{2}\hbox {CuO}_4$^18^, particularly the measurements with inelastic neutron scattering (INS)^12,13^. In past decades, much experimental evidence showed that resonant inelastic x-ray scattering (RIXS) is a complementary tool to INS for probing magnetic fluctuations^19–25^. Theoretically, direct spin-flip scattering is allowed in *L*-edge RIXS^26–32^; Jia et al. showed that Cu *L*-edge RIXS provides access to the spin dynamical structure factor if the x-ray polarization is fully analyzed^30,31^. The pioneering work of Braicovich et al. on Cu *L*-edge RIXS has successfully measured the single-magnon dispersion of cuprates^19–21^. With crossed polarizations between the incident and scattered x-rays, RIXS detects spin fluctuations directly, requiring a much smaller sample volume and enabling easy detection of high-energy excitations, as opposed to INS. For some cases, it is even more powerful than INS as it also probes charge, orbital, and lattice excitations and the effect of the matrix element gives rise to a possibility of tuning the cross-section involved with different scattering channels; for example, see Refs. [^22,23,33–41^]. However, separating bimagnon or even-order excitation from a single magnon is delicate in Cu $L_3$-edge RIXS^42^. Using exact diagonalization (ED) calculations on a single-band Hubbard model, Tsutsui and Tohyama^32^ revealed that Cu *L*-edge RIXS can measure bimagnon excitations of cuprates at half filling if the incident photon energy is tuned to be above the $L_3$ absorption energy in the order of *J*.

In the present article, we combined RIXS measurements and ED calculations on the single-band Hubbard model to study the magnetic excitations of $\hbox {La}_{2}\hbox {CuO}_4$, which represents a paradigm for quantum magnetism and is a parent compound of cuprate superconductors. For the first time, we demonstrate experimentally that bimagnon excitation in RIXS is enhanced if the incident x-ray energy is slightly above the absorption edge. The measured bimagnon dispersion of the in-plane momentum transfer ${\bf{q}}_{\Vert }$ along the Cu-O bond direction agrees well with the ED calculations. This paper is organized as follows. We begin by describing the experimental and computational methods, including Cu *L*-edge RIXS measurements and ED calculations. Next, we present the Cu *L*-edge RIXS results, focusing on their dependence on incident energy and momentum. Finally, we discuss the observed bimagnon dispersions.

**Fig. 1:**
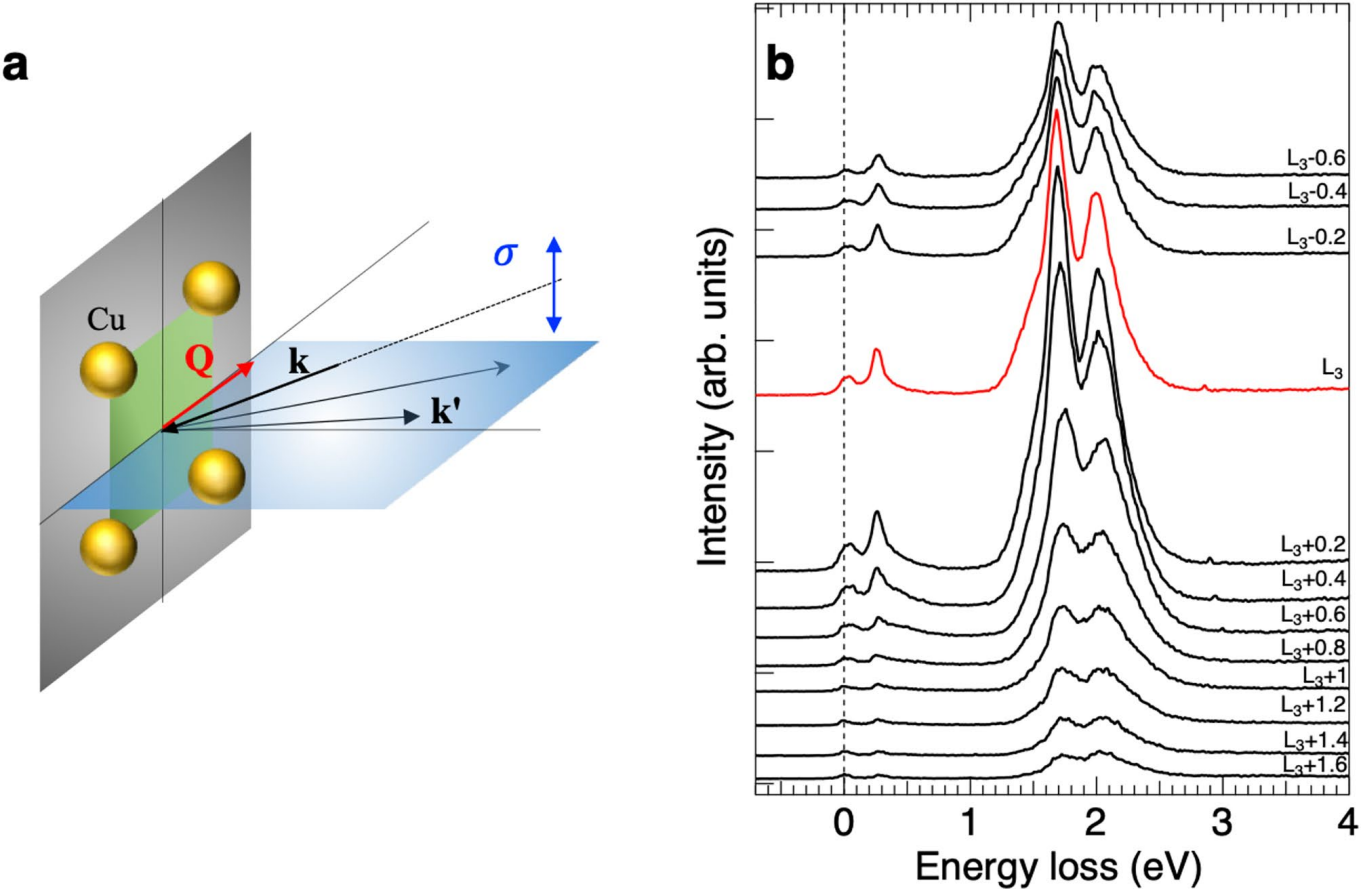
Incident-energy-dependent Cu *L*-edge RIXS of $\hbox {La}_{2}\hbox {CuO}_4$. (**a**) Scattering geometry of the RIXS measurements. The scattering plane is defined by the incident and scattered wave vectors, **k** and ${\bf{k}}^{\prime }$, respectively. The projection of the momentum transfer onto the $\hbox {CuO}_2$ plane, ${\bf{q}}_{\Vert }\equiv ~{\bf{k}}-{\bf{k}}^\prime$ is along the anti-nodal direction $(\pi , 0)$. (**b**) RIXS spectra of ${\bf{q}}_\Vert =(0.5\pi , 0)$ for various incident energies across Cu $L_3$-edge. The red spectrum corresponds to the $L_{3}$ peak in XAS. Spectra are plotted with a vertical offset for clarity.

## Results

### Incident-energy-dependent RIXS

Figure 1 shows incident-energy-dependent RIXS spectra at Cu $L_3$-edge with ${\bf{q}}_{\Vert } = (\pi , 0)$ in units of 1/*a*, with which all momentum transfers are expressed throughout the paper; here, *a* is the lattice constant. In the RIXS process of cuprates, a 2*p* electron is excited by an incident x-ray of energy $\omega _\textrm{i}$ to the unoccupied upper Hubbard band, followed by the decay of an electron in the valence 3*d* band to the 2*p* core hole to form a *dd* excitation. Figure 1**b** plots a series of RIXS spectra from $\hbox {La}_{2}\hbox {CuO}_4$, when the incident energy $\omega _\textrm{i}$ is tuned to the $L_3$ absorption edge, the intensity of the *dd* feature reaches a maximum. The energy and spectral line shape of the *dd* excitation reflect the local electronic structure. We observed *dd*-excitation features at energies $\sim$ 1.5 eV, 1.7 eV, and 2.0 eV.

**Fig. 2:**
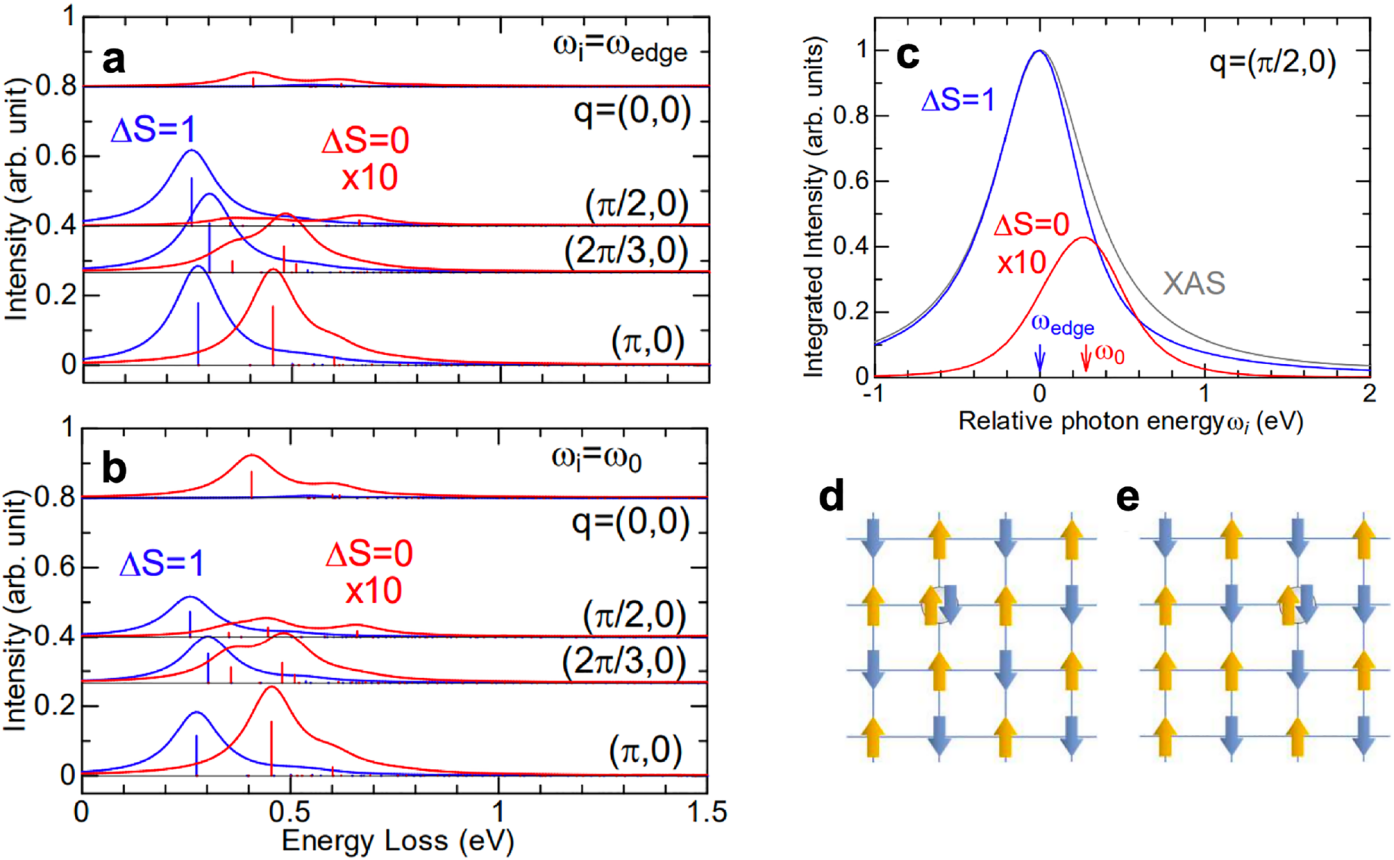
Calculated Cu $L_3$-edge RIXS spectra with the spin-flip and non-spin-flip processes. (**a**,**b**) RIXS spectra for various in-plane momenta ${\bf{q}}_{\Vert }$ and photon energy $\omega _i$ tuned to the absorption edge $\omega _\textrm{edge}$ and $\omega _0\sim 0.28$ eV with which the integrated intensity for $\Delta S=0$ is maximum. RIXS spectra are presented by the calculated spectral weights, i.e., the $\delta$-functions shown by the vertical thin solid lines, after convolution with Lorentzian broadening of 0.2*t*. Spectra are vertically offset for clarity. (**c**) Photon energy $\omega _i$ dependence of the integrated RIXS intensity with $\Delta S=0$ (red) and 1 (blue) at ${\bf{q}}_{\Vert }=(\frac{\pi }{2},0)$, as well as the calculated XAS (gray). $\omega _\textrm{edge}$ and $\omega _0$ are marked by the blue and red arrows, respectively. (**d**)  Spin configurations with the largest weights in the XAS edge state indicated by the blue arrow in (**c**). The core holes in the RIXS intermediate state are created in the down-spin sublattice. (**e**)  Spin configurations with the largest weights in an eigenstate near the energy indicated by the red arrow in (**c**). The core holes in the RIXS intermediate state are created in the up-spin sublattice.

In addition, collective excitations such as phonon, magnon and plasmon^21,36,37,43,44^ excitations can be induced by Cu *L*-edge RIXS, accompanying the *dd* excitations. In the RIXS intermediate state, the 2*p* core hole is with strong spin-orbit coupling. As the spin is no longer a good quantum number in the 2*p* orbital, *L*-edge RIXS permits excitation involved with a spin flip. The matrix elements of the operators ${S^{j}_{\bf{q}}}$ and ${N^{j}_{\bf{q}}}$ govern the RIXS excitations with the change of total spin by one ($\Delta S =1$) and no spin change ($\Delta S =0$), respectively. If the polarization of the incident x-ray is selected, one can in principle analyze the polarization of the scattered x-ray to separate RIXS excitations with $\Delta S=1$ and 0.

Before presenting our RIXS data, we first discuss the resonance effect of the magnon and bimagnon excitations in RIXS through ED calculations. For the half-filling, a bimagnon-type operator dominates the non-spin-flip process^32^. Figure 2a, b compare the calculated RIXS excitations with $\Delta S=1$ and 0 at selected in-plane momentum transfers for the incident photon energy $\omega _i$ tuned to the $L_3$ absorption edge and 0.27 eV above, defined as $\omega _0$. The comparison indicates that, although the intensity is weak, the bimagnon RIXS excitation has higher energy than that of a single magnon, roughly by 0.2 eV. Figure 2c plots calculated intensities of absorption spectrum (XAS) and RIXS with $\Delta S=1$ and 0 as a function of the incident x-ray energy. The bimagnon RIXS resonates at an incident photon energy 0.27 eV higher than the XAS edge and the single-magnon resonance. To conceptually explain the reason that the bimagnon excitation is enhanced at energy slightly above the absorption edge, we depict the spin configurations around the $3d^{10}$ site of the XAS final state, i.e., the intermediate state of RIXS, in Fig. 2d, e for the incident energy tuned to the absorption edge and $\omega _0$, respectively.

**Fig. 3:**
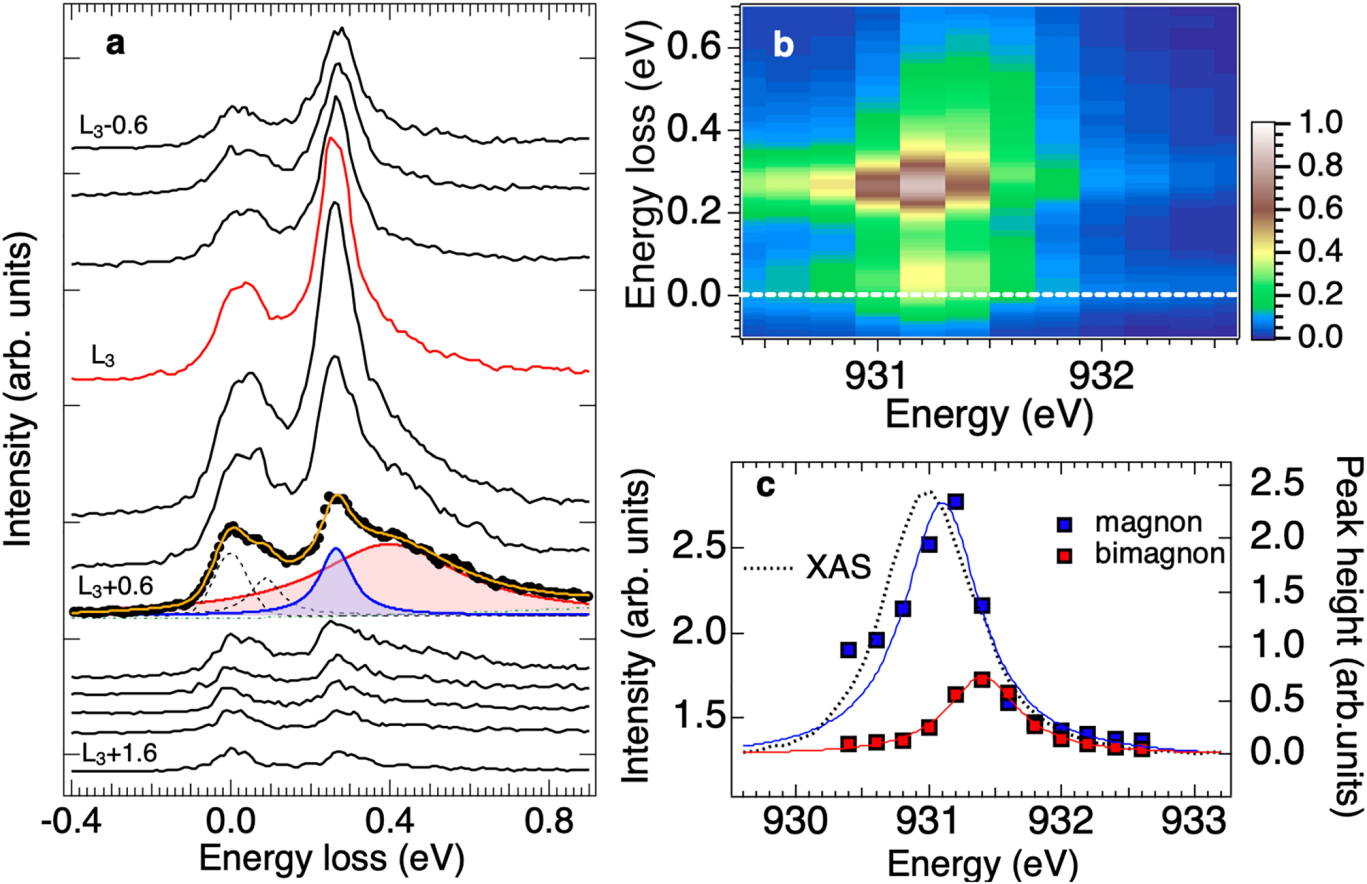
Spin excitation of $\hbox {La}_{2}\hbox {CuO}_4$ in incident-energy-dependent Cu *L* -edge RIXS. (**a**) Cu *L*-edge RIXS spectra of ${\bf{q}}_{\Vert }$ $=(0.5\pi , 0)$ for various incident energies across the Cu $L_3$-edge. Spectra are plotted with a vertical offset for clarity. The spectral components of magnon and bimagnon from the curve fitting to the RIXS spectrum induced by x-ray energy of $L_{3}+0.6$ eV are plotted in colors. The dotted lines plot the elastic and the phonon components. See the supplementary material for the curve-fitting details. (**b**) Two-dimensional RIXS intensity map measured across the Cu $L_3$-edge. (**c**) The peak height of the magnon (blue square) and bimagnon (red square) components from the fitting as a function of incident photon energy across the $L_3$-edge. Solid lines plot Lorentizian distribution curves to highlight the resonance along with XAS (gray dashed lines).

In the initial state of the XAS and RIXS, there are configurations in which an up spin and a down spin are exchanged from the complete Néel spin configuration because of the quantum fluctuations. Figure 2d, e illustrate the spin configurations of the RIXS intermediate state (equivalent to the XAS final state) in two distinct scenarios. Figure 2d shows the excitation of an up-spin electron from the 2*p* core level to a down-spin site on the down-spin sublattice in the Néel configuration. In contrast, Fig. 2e depicts an alternative channel, in which an up-spin electron is excited to an exchanged down-spin site on the up-spin sublattice in the fluctuated configuration. The spin arrangements surrounding the core site differ between these two cases, resulting in an energy difference on the order of *J*. This energy difference can naturally lead to bimagnon excitations once the up-spin electron and the core hole are removed via photon emission. That is, X-rays with additional energy on the order of *J* can enhance the bimagnon spectral weight. Therefore, the bimagnon excitation can enhance through a state in energy higher than the XAS edge state.

### Momentum-dependent RIXS

We used RIXS with incident x-ray energies tuned across the Cu $L_3$-edge absorption of energy denoted $L_3$ to study the dispersion of bimagnons. Figure 3a, b show the magnified RIXS spectra and intensity map of $\hbox {La}_{2}\hbox {CuO}_4$, respectively. In agreement with previous results^19,20,45^, phonon and magnon excitations exist for energies smaller than 100 meV and around 280 meV, respectively. The energy of the sharp and well-defined magnon excitation is independent of the incident photon energy; it is a Raman-like excitation. As plotted in Fig. 3a, there is a pronounced spectral feature centered at 0.4 eV on the high-energy side of the magnon excitation for the incident photon energy at $L_{3}$+0.4 eV, in agreement with the theoretical prediction on the bimagnon RIXS feature^32^. Figure 3c displays the plot of magnon and bimagnon intensities versus the incident photon energy to show their different resonance effects. The bimagnon resonates at an incident photon energy higher than the magnon by 0.3 eV.

**Fig. 4:**
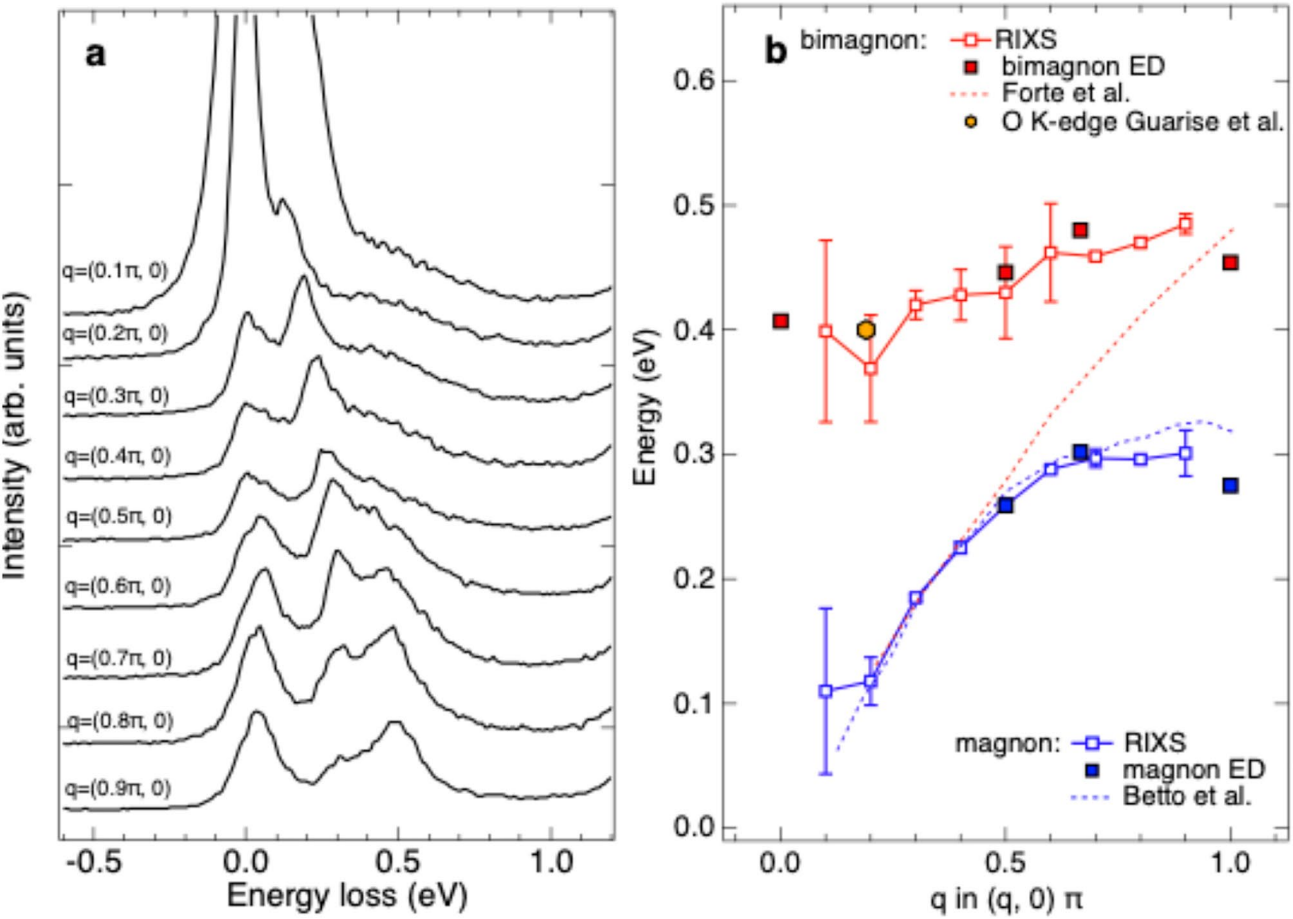
Bimagnon dispersion of $\hbox {La}_{2}\hbox {CuO}_4$ from momentum-dependent Cu *L*-edge RIXS. (**a**) Cu *L*-edge RIXS spectra for various in-plane momentum transfers with incident energy tuned to $L_3$+0.6 eV. RIXS spectra are after correction for self-absorption and plotted with vertical offsets for clarity. (**b**) Energies of magnon and bimagnon extracted from RIXS, along with the energy positions of the maximum intensities in the calculated RIXS spectra with $\Delta S=0$ (red squares) and 1 (blue squares). Details of self-absorption correction and curve fitting analysis for determining the energy positions are presented in the supplementary material. For comparison, the energy positions of the single magnon from Betto et al.^46^ and the bimagnon from Forte et al.^47^ and Guarise et al.^48^ are also plotted in (**b**).

The combined results of incident-photon-energy dependent RIXS measurements and ED calculations provide us with an opportunity to study the dynamics of magnon excitation. We set the incident photon energy to 0.6 eV above the $L_3$ absorption edge for measuring the dispersion of bimagnon. Figure 4a shows momentum-dependent RIXS spectra. After the curve fitting analysis shown in the supplementary material, we found that the measured bimagnon disperses from 0.4 eV for ${\bf{q}}_{\Vert } = (0, 0)$ toward 0.5 eV for ${\bf{q}}_{\Vert } = (\pi , 0)$ as plotted in Fig. 4b.

## Discussion

**Fig. 5:**
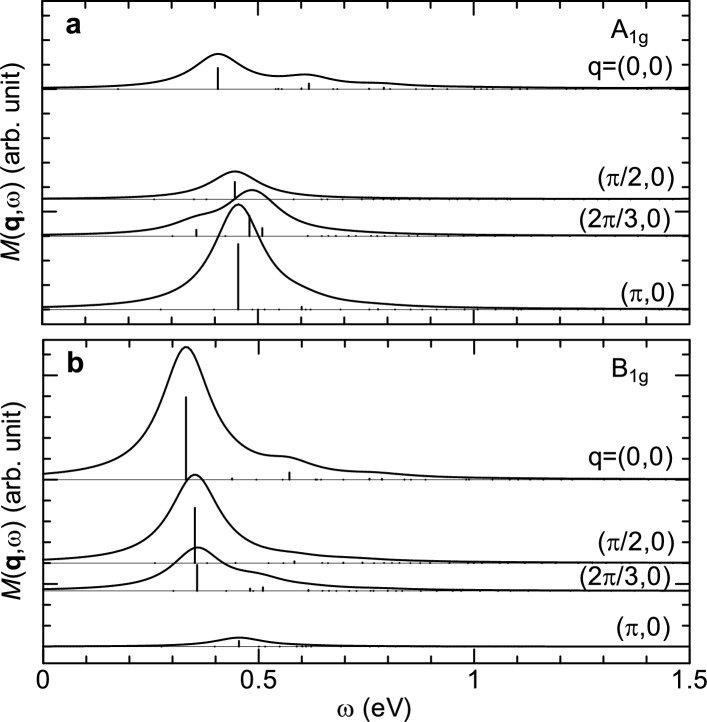
Calculated bimagnon correlations. (a,b) Bimagnon correlations $M(\textbf{q}, \omega )$ with selected $\textbf{q}$ for $A_{1g}$ and $B_{1g}$, respectively. $M(\textbf{q}, \omega )$ were calculated through $\sum _{f}|\langle {f}|M_{\bf{q}}^{\pm }|0\rangle |^2\delta (\omega -E_{f}+E_{0})$, where $|0\rangle$ and $|f\rangle$ represent, respectively, the ground and final states of energy $E_0$ and $E_f$. The spectra are presented by the calculated spectral weights, i.e., the $\delta$-functions shown by the vertical thin solid lines, after convolution with Lorentzian broadening of 0.2*t*. Spectra are vertically offset for clarity.

To further understand the bimagnon dynamics of $\hbox {La}_{2}\hbox {CuO}_4$, we calculated the dynamical bimagnon correlation function, which dominates the non-spin-flip process of the half-filling through its matrix elements^32^. The dynamical bimagnon correlation function is given by a correlation function of the operator $M_{\bf{q}}^{\pm }=\sum _{\bf{k}}(\cos k_{x}{\pm }\cos k_{y}){\bf{S}}_{\bf{k+q}}\cdot {\bf{S}}_{\bf{-k}}$, where $+~(-)$ corresponds to $A_{1g}~(B_{1g})$ mode; ${\bf{S}}_{\bf{k+q}}$ and ${\bf{S}}_{\bf{-k}}$ are the spins of wave vectors ${\bf{k+q}}$ and ${\bf{-k}}$, respectively^32^. Figure 5 shows the calculated ${\bf{q}}$-dependent dynamical bimagnon correlation function for $A_{1g}$ and $B_{1g}$ modes. As shown in Fig. 5a, the energy positions of $A_{1g}$ mode indicated by $\delta$-functions have much better correspondence to those in the calculated RIXS spectra with $\Delta S=0$ in Fig. 2b. In addition, as shown in Fig. 5b, the ED calculations reveal that the bimagnon spectral weight of $B_{1g}$ mode decreases drastically as ${\bf{q}}_{\Vert }$ is increased toward $(\pi , 0)$, in contrast to our measurement plotted in Fig. 4a. This indicates that the bimagnon of $\hbox {La}_{2}\hbox {CuO}_4$ detected by Cu *L*-edge RIXS is predominantly of $A_{1g}$ mode.

The observed bimagnon dispersion presented in Fig. 4b shows a flat-like feature, in sharp contrast to a monotonic and steep dispersion predicted by calculations of the Heisenberg model with only the nearest neighbor exchange interaction^47^. Our observation is consistent with the dispersion in the ED calculations on the Hubbard model. The coherent motion of electrons beyond nearest-neighbor sites and higher-order spin couplings are naturally included in the Hubbard model. This means that the effect of the higher order terms of *t*/*U* such as the cyclic interactions is important for the bimagnon dynamics.

The combination of our Cu $L_3$-edge RIXS measurements and ED calculations provides a simple scheme to measure the energy dispersion of bimagnon without resorting to polarization analysis on the scattered x-rays. The incident light was of $\sigma$ polarization, and scattered light of both $\sigma$ and $\pi$ polarization was detected. If the RIXS spectra are recorded at the $L_3$ absorption edge, both channels of $\Delta S = 0$ and $\Delta S = 1$ will be included and the bimagnon excitation will be strongly mixed with single-magnon excitations and the continuum of odd-number magnons^46,49,50^. For the incident x-ray tuned to be above the absorption edge, the bimagnon weight is enhanced. As illustrated in Fig. 2d,e, dominant spin configurations in the RIXS intermediate state having one doubly occupied core-hole site surrounded by spin background are different between RIXS transitions at and above the absorption edge. The latter is more suitable for a coupling to the bimagnon spin configuration in the RIXS final state. This explains why the bimagnon intensity is enhanced. Therefore the measured bimagnon dispersion is more reliable than those extracted from RIXS data recorded with x-rays tuned to the absorption edge.

In short, our RIXS measurements agree well with ED calculations and the results unravel the bimagnon dispersion of $\hbox {La}_{2}\hbox {CuO}_4$ although the measurement method is straightforward. The effect of the higher-order spin couplings beyond nearest-neighbor sites is important for understanding the bimagnon dynamics of cuprates.

## Methods

### Sample synthesis

The $\hbox {La}_{2}\hbox {CuO}_4$ single crystal was grown by the traveling-solvent floating zone method^51,52^. After growth, the crystals were annealed appropriately to remove oxygen defects. The oxygen content was tuned to be $4.000{\pm }0.001$ following Ref.^53^. For more details on the crystal growth and characterization, see Ref.^51^.

### RIXS measurements

We conducted RIXS measurements using the AGM-AGS spectrometer of beamline 41A at Taiwan Photon Source^54^. This RIXS beamline has been constructed based on the energy-compensation principle of grating dispersion. The crystallographic axes of the $\hbox {La}_{2}\hbox {CuO}_4$ crystal were precisely aligned with x-ray Laue diffraction and using a special sample holder with tilting adjustment. Before RIXS measurements, the $\hbox {La}_{2}\hbox {CuO}_4$ sample was cleaved in air and then mounted on a 3-axis in-vacuum manipulator through a load-lock system. X-ray absorption spectra were measured using a photodiode in the fluorescence yield mode. The sample was cooled to 30 *K* with liquid helium and RIXS measurements at the Cu *L*-edge were recorded with $\sigma$-polarized incident x-rays for various incident energies with an energy resolution of $\sim 90$ meV FWHM at Cu $L_3$-edge and in-plane wave-vector changes along the anti-nodal direction. The momentum resolution was $\sim 0.006~\text{\AA }^{-1}$ for the *q*-dependent measurements.

### Cu *L*-edge RIXS calculations

We calculated Cu *L*-edge RIXS spectra using the Lanczos-type diagonalization method on a single-band Hubbard model with periodic $4\times 4$- and $\sqrt{18}\times \sqrt{18}$-site square-lattice clusters. The model Hamiltonian was

1 $$\begin{aligned} H_{3d} = -t \sum _{i\delta \sigma }c^{\dag }_{i\sigma }c_{(i+\delta )\sigma } -t'\sum _{i\delta '\sigma }c^{\dag }_{i\sigma }c_{(i+\delta ')\sigma } + U\sum _{i}n_{i\uparrow }n_{i\downarrow }, \end{aligned}$$

where $c^{\dag }_{i\sigma }$ is the creation operator of an electron with spin $\sigma$ at site *i*. The number operator $n_{i\sigma }$ is $c^{\dag }_{i\sigma }c_{i\sigma }$, and $i+\delta$ ($i+\delta '$) represents the four first (second) nearest-neighbor sites around site *i*. The hopping energy *t* was a fitting parameter to match the values of the single-magnon energy at $(\frac{\pi }{2}, 0)$. Here the model parameters were set as the second-nearest-neighbor hopping $t'/t=-0.25$, on-site Coulomb interaction $U/t=10$, and $t=0.35$ eV. For an energy loss $\Delta \omega$, the RIXS spectra with $\Delta S=1$ and 0 are given by

2 $$\begin{aligned} I_{\bf{q}}^{\Delta S=1}(\Delta \omega )= & \sum _{f}|\langle {f}|{S^{j}_{\bf{q}}}|0\rangle |^2\delta (\Delta \omega -E_{f}+E_{0}),\end{aligned}$$

3 $$\begin{aligned} I_{\bf{q}}^{\Delta S=0}(\Delta \omega )= & \sum _{f}|\langle {f}|{N^{j}_{\bf{q}}}|0\rangle |^2\delta (\Delta \omega -E_{f}+E_{0}), \end{aligned}$$

where $|0\rangle$ and $|f\rangle$ represent, respectively, the ground and final states of energy $E_0$ and $E_f$. The operators $S_{\bf{q}}^{j}$ and $N_{\bf{q}}^{j}$ are defined by $S_{\bf{q}}^{j}=(B_{{\bf{q}}\uparrow \uparrow }^{j}-B_{{\bf{q}}\downarrow \downarrow }^{j})/2$ and $N_{\bf{q}}^{j}=(B_{{\bf{q}}\uparrow \uparrow }^{j}+B_{{\bf{q}}\downarrow \downarrow }^{j})$ with

4 $$\begin{aligned} B_{{\bf{q}}\sigma '\sigma }^{j}=\sum _{l}e^{-i{\bf{q}}{\cdot } {\bf{R}}_l}c_{l\sigma '}\frac{1}{\omega _i - H^{j}_l + E_0 +i\Gamma }c^{\dagger }_{l\sigma }, \end{aligned}$$

where ${\bf{R}}_l$ is the position vector at site *l*, $H^j_l=H_{3d}-U_c\sum _\sigma { n_{l\sigma }}+\varepsilon _j$ with $U_c$ and $\varepsilon _j$ being the Cu 2*p*-3*d* Coulomb interaction and energy level of Cu 2*p* core hole at site *l*, and *j* is the total angular momentum of the Cu 2*p* hole with $j=3/2$ for the present $L_3$ edge. The parameter for the core-hole lifetime was set as $\Gamma /t=1$, $U_c/t=12$, and the value of $\varepsilon _j$ was determined to match the energy of the edge in the XAS. The polarization dependence of the incident and scattered photons was not included in the calculation of RIXS spectral weight. See Ref.^32^ for other calculation details. Correspondingly, the Cu *L*-edge XAS spectrum is also defined in Ref.^32^, where $\Gamma _\textrm{XAS}=\Gamma$.

## Supplementary Information

Supplementary Information.

## Data Availability

All data generated or analysed during this study are included in this published article [and its supplementary information files].
