# Supplementary material for: Bimagnon dispersion of La2CuO4 probed by resonant inelastic X-ray scattering
Source: Sci Rep. 2025 Oct 1;15:34183. doi: 10.1038/s41598-025-15435-5 (PMC12488913; doi:10.1038/s41598-025-15435-5)
Supplement: Supplementary file 1 — Supplementary Information. [file 41598_2025_15435_MOESM1_ESM.pdf]

Supplementary Information for

"Bimagnon dispersion of La_2CuO_4 probed by resonant inelastic x-ray scattering"

Curve-fitting data analysis on Cu L-edge RIXS spectra

A. Singh, H. Y. Huang, K. Tsutsui, T. Tohyama, S. Komiya, J. Okamoto
C. T. Chen, A. Fujimori, D. J. Huang*

* Correspondence to:
djhuang@nsrrc.org.tw

June 6, 2025

Figure S1 shows the Cu L_3 edge x-ray absorption spectroscopy (XAS) spectrum of La_2CuO_4 measured with σ polarization by using the total electron yield method. The red triangles represent the energy positions selected for the resonant inelastic x-ray scattering (RIXS) measurements. We measured the RIXS spectra for incident energies from L_3-0.6 eV to L_3+1.6 eV in the 0.2 eV step, where L_3 denotes the Cu L_3-edge absorption energy of the XAS shown in Fig. S1. For the momentum-dependent RIXS spectra I_{rixs}, the self absorption effect of the raw data I_{raw} was corrected with the following the equation (Ref [1, 2]),

$$I_{\mathrm{rixs}} \propto I_{\mathrm{raw}} \frac{\sin(\Omega - \theta)}{\sin(\Omega - \theta) + \sin\theta} , \tag{1}$$

where Ω and θ are the scattering angle and the incident angle of the x-ray from the sample surface. The absorption coefficients before and after scattering were assumed to be unchanged.

To identify the magnon and bimagnon energy positions, we performed a least-squares curve fitting of the RIXS spectra by using four components: one Gaussian profile for elastic scattering, and three Lorentzian profiles for phonon, magnon, and bimagnon excitations. We first fitted the RIXS spectrum measured at L_3+0.6 eV, where the magnon and bimagnon peaks are well resolved. For the fitting of other incident energies, the peak positions of the magnon and bimagnon components were kept fixed, while their peak widths were varied. Figure S2 shows the measured and fitted energy-dependent RIXS spectra along with the fitted components. Similarly, we performed the curve fitting of the momentum-dependent RIXS spectra with four components as shown in Fig. S3.

For the magnetic excitations of La_2CuO_4 measured with Cu L-edge RIXS, there exists a continuum mode associated with fractional spin excitations [3]. To estimate the uncertainty of bimagnon energy, we also fitted the momentum-dependent RIXS spectra with this additional mode included. Figure S4 shows the measured and fitted RIXS spectra including fractional spin excitations. Further, we compared the magnon and bimagnon dispersions from these two

fitting procedures, as shown in Fig. S5, and obtained the variation in the fitted energy of the two procedures for each q. The error bars shown in Fig. 4 of the main paper are expressed in terms of the root mean square of individual fitted errors and the variation from the curve fitting.

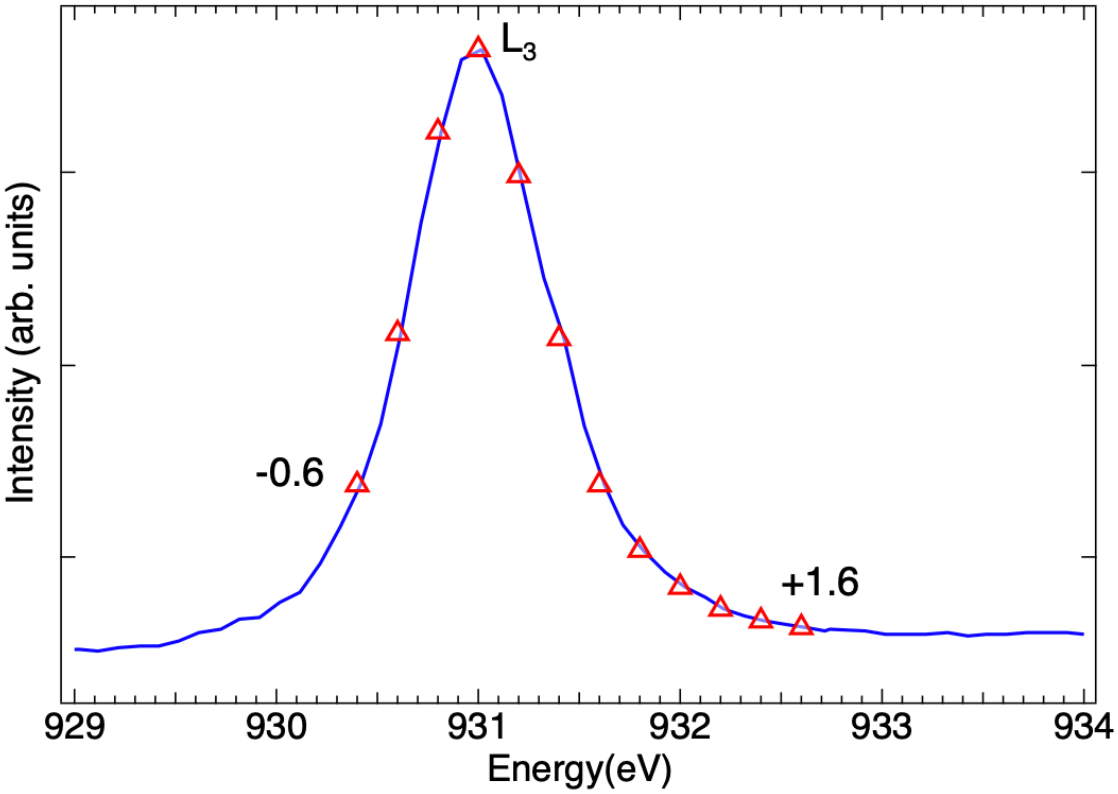

Figure S1: The Cu L_3-edge XAS spectrum of La_2CuO_4 measured with σ polarization by using the total electron yield method. The XAS peak energy is denoted as L_3, and the red triangles indicate the energies selected for the RIXS measurements.

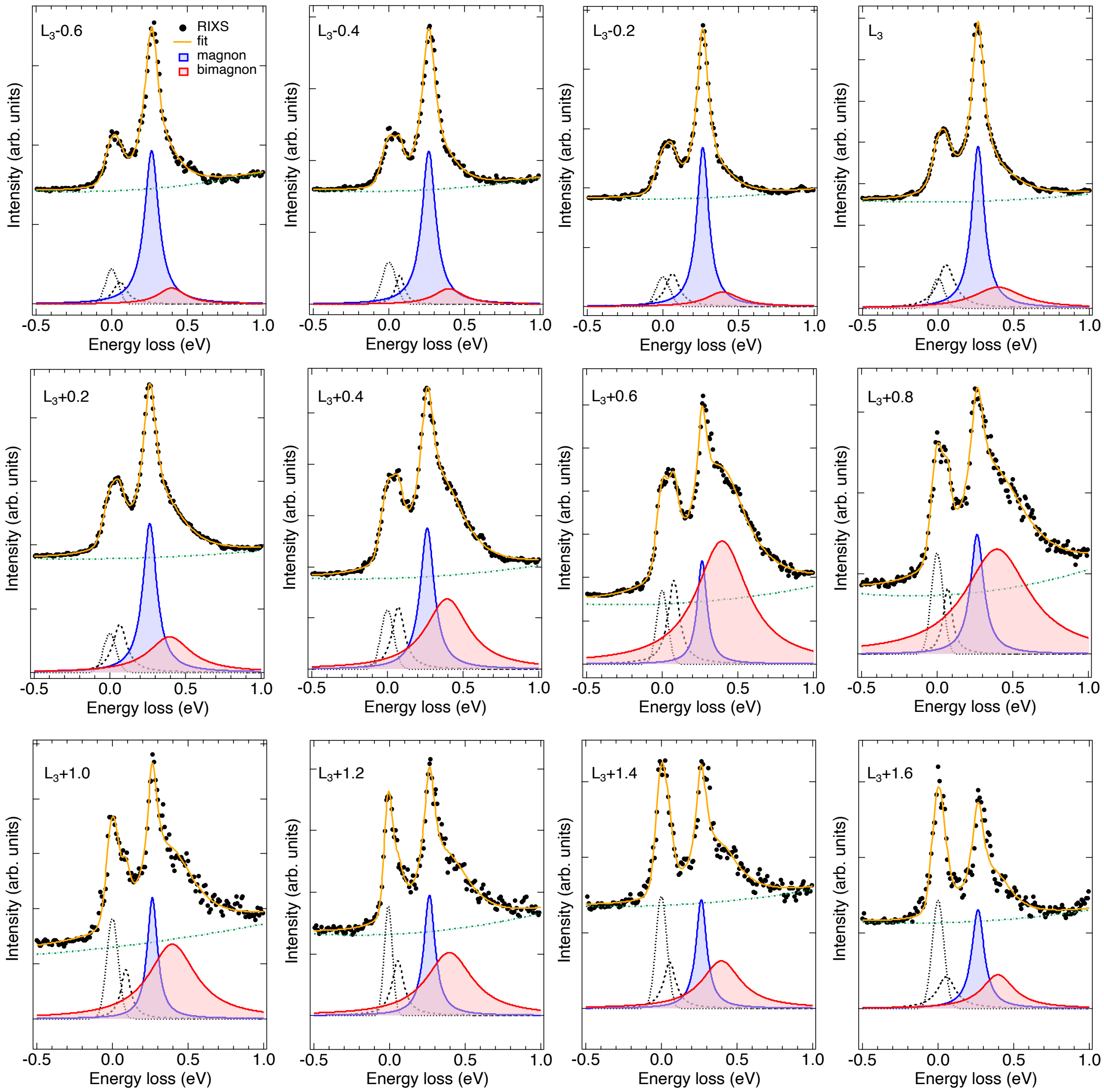

Figure S2: Energy-dependent RIXS spectra and fitted results of La_2CuO_4. RIXS spectra are measured for $\mathbf{q}_{\parallel} = (\frac{\pi}{2}, 0)$ at 25 K. The solid black circles represent the measured data, and the solid orange lines represent the fitted curves. For each spectrum, the fitted components include an elastic peak (black dashed line), a phonon peak (black dotted line), a magnon peak (blue) and a bimagnon peak (red); the last two components are shaded in color. The background is plotted with a green dashed line.

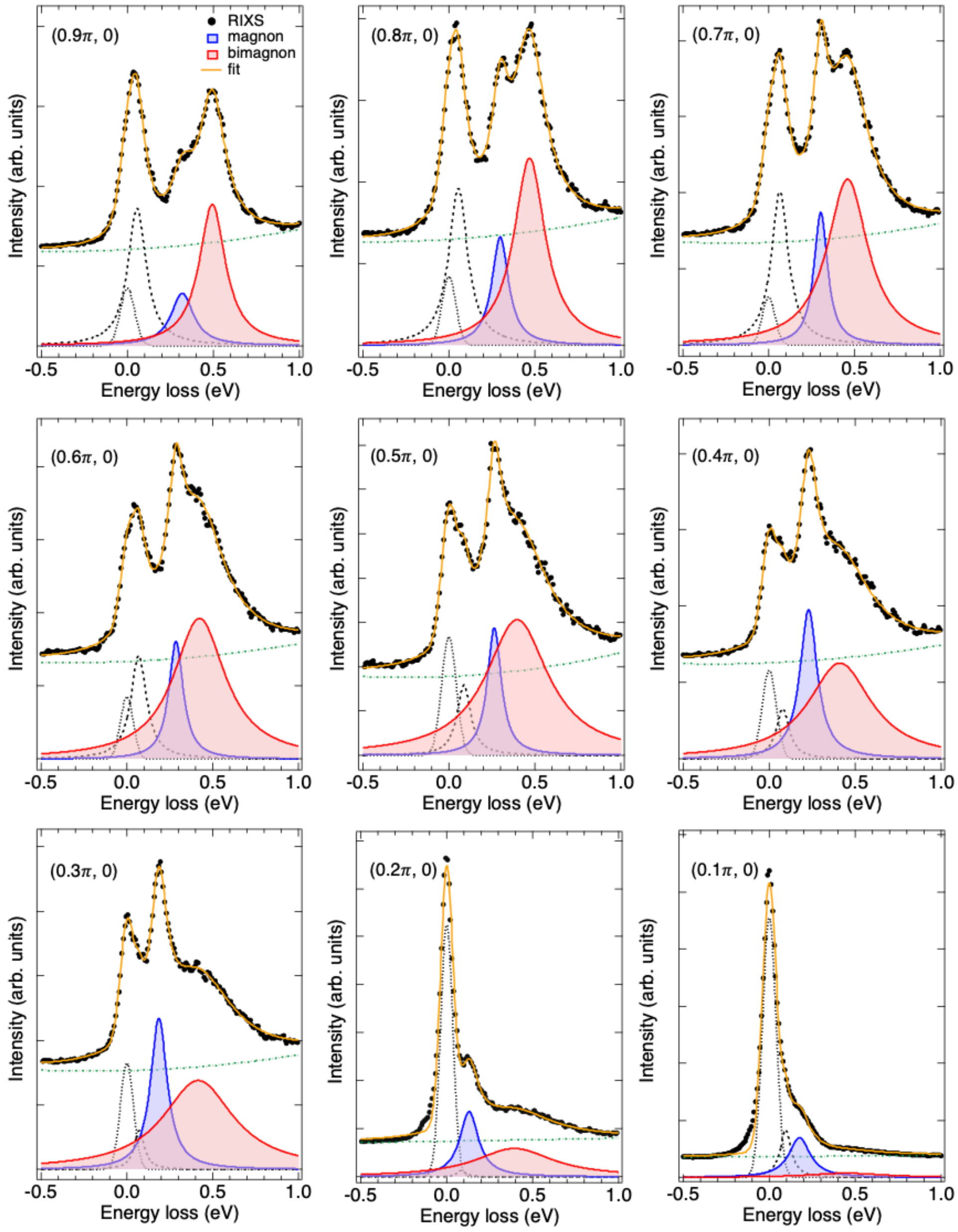

Figure S3: Momentum-dependent RIXS spectra and fitted results of La_2CuO_4. RIXS spectra are measured for $E = L_3 + 0.6$ eV at 25 K. Figure legends are the same as those in Fig. S2.

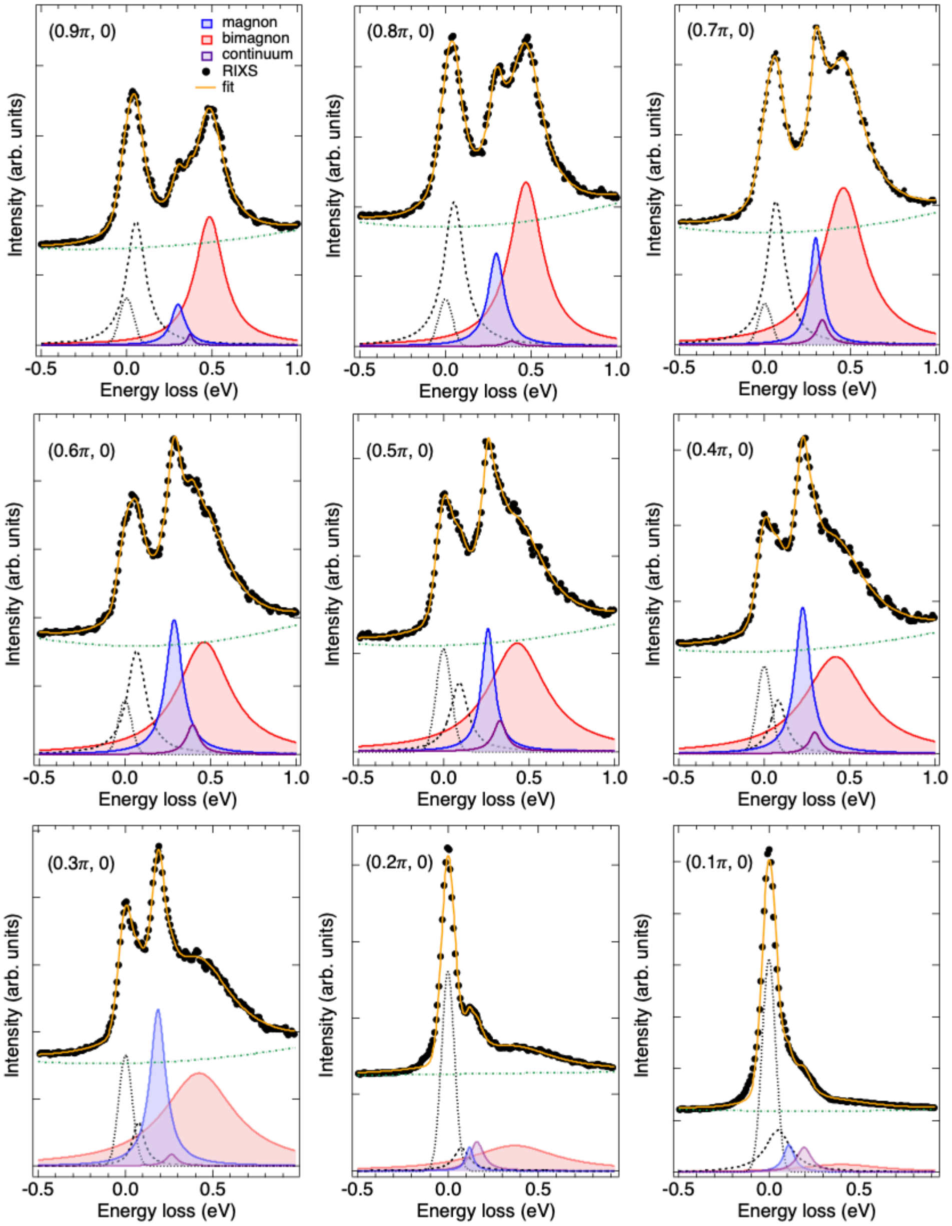

Figure S4: Momentum-dependent RIXS spectra and fitted results of La_2CuO_4. RIXS spectra and figure legends, except for the continuum mode (purple), are the same as those in Fig. S3.

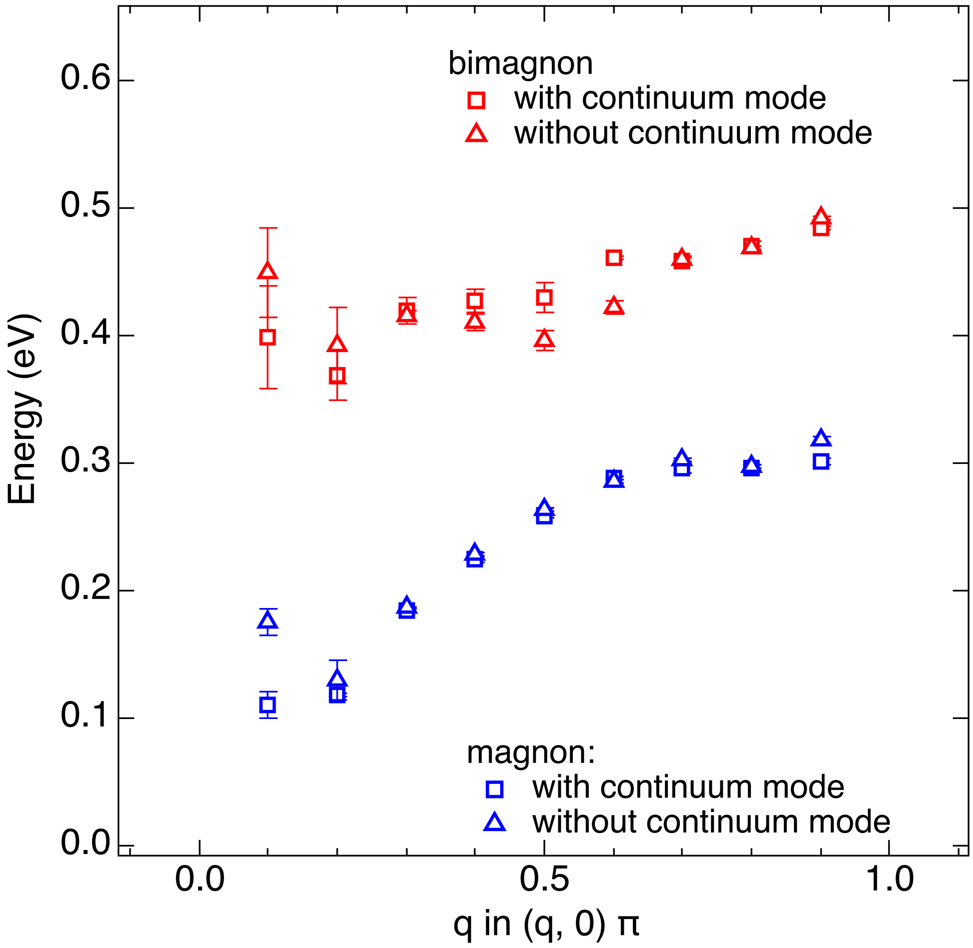

Figure S5: Magnon and bimagnon dispersion, obtained through the curve fitting from two different methods. Red and blue squares correspond to the positions obtained with including the continuum mode, while red and blue triangles correspond to the positions obtained without the continuum mode.

References

[1] Achkar, A. J. *et al.* Bulk sensitive x-ray absorption spectroscopy free of self-absorption effects. *Phys. Rev. B* **83**, 081106 (2011).

[2] Kang, M. *et al.* Resolving the nature of electronic excitations in resonant inelastic x-ray scattering. *Phys. Rev. B* **99**, 045105 (2019).

[3] Martinelli, L. *et al.* Fractional Spin Excitations in the Infinite-Layer Cuprate $CaCuO_2$. *Phys. Rev. X* **12**, 021041 (2022).
